# Comparison of two rapid automated analysis tools for large FTIR microplastic datasets

**DOI:** 10.1007/s00216-023-04630-w

**Published:** 2023-03-20

**Authors:** Sonya R. Moses, Lisa Roscher, Sebastian Primpke, Benedikt Hufnagl, Martin G. J. Löder, Gunnar Gerdts, Christian Laforsch

**Affiliations:** 1 Department of Animal Ecology I and BayCEER, University of Bayreuth, Universitätsstr. 30, 95440 Bayreuth, Germany; 2 Alfred-Wegener-Institute Helmholtz Centre for Polar and Marine Research, Biologische Anstalt Helgoland, Kurpromenade 201, 27498 Helgoland, Germany; 3 Institute of Chemical Technologies and Analytics, Vienna University of Technology, A 1060 Vienna, Austria; 4 Purency GmbH, Walfischgasse 8/34, A 1010 Vienna, Austria

**Keywords:** Automated microplastic analysis, FTIR, siMPle, Bayreuth Particle Finder (BPF), Freshwater samples, Seawater samples

## Abstract

One of the biggest issues in microplastic (MP, plastic items  <5 mm) research is the lack of standardisation and harmonisation in all fields, reaching from sampling methodology to sample purification, analytical methods and data analysis. This hampers comparability as well as reproducibility among studies. Concerning chemical analysis of MPs, Fourier-transform infrared (FTIR) spectroscocopy is one of the most powerful tools. Here, focal plane array (FPA) based micro-FTIR (µFTIR) imaging allows for rapid measurement and identification without manual preselection of putative MP and therefore enables large sample throughputs with high spatial resolution. The resulting huge datasets necessitate automated algorithms for data analysis in a reasonable time frame. Although solutions are available, little is known about the comparability or the level of reliability of their output. For the first time, within our study, we compare two well-established and frequently applied data analysis algorithms in regard to results in abundance, polymer composition and size distributions of MP (11–500 µm) derived from selected environmental water samples: (a) the siMPle analysis tool (systematic identification of MicroPlastics in the environment) in combination with MPAPP (MicroPlastic Automated Particle/fibre analysis Pipeline) and (b) the BPF (Bayreuth Particle Finder). The results of our comparison show an overall good accordance but also indicate discrepancies concerning certain polymer types/clusters as well as the smallest MP size classes. Our study further demonstrates that a detailed comparison of MP algorithms is an essential prerequisite for a better comparability of MP data.

**Supplementary Information:**

The online version contains supplementary material available at 10.1007/s00216-023-04630-w.

## Introduction

While the microplastic (MP, plastic items  <5 mm) contamination of the environment is constantly growing, MP has meanwhile been detected in all ecosystems reaching from aquatic [1–3] to aerial [4–6] to terrestrial systems [7–9] and has also been found in remote areas far from population centres such as Arctic ice [10, 11] and remote mountain lakes [12, 13] but also in biota [14, 15]. For an accurate risk assessment reliable analytical methods are thus urgently required to appropriately identify MP isolated from environmental matrices qualitatively and quantitatively [16]. The field of MP research arose from MP detected in coastal waters in the 1970s, initially focusing on rather large MPs (sampled with plankton tow nets (mesh size: 333 µm)) [17, 18]. Due to their size, manual handling of prospective large MP items for analysis is possible and is common practice. Commonly applied methods for larger MP particles are visual identification [19], Nile red staining [20, 21], the hot needle method [22] or through attenuated total reflectance (ATR)-Fourier transform-infrared (FTIR) spectroscopy [23–25] which allows for chemical identification of the respective polymer. However, most of these methods are prone to human bias, leading to an over- or underestimation of the MP abundance. While analysis with ATR-FTIR also requires a manual preselection of prospective MP and is thus also susceptible to bias, the chemical identification of polymers is reliable which makes this method favourable for the analysis of larger MP.

Recent studies have shown that the abundance of small MP (S-MP,  <500 µm) in the environment is much higher and thus should not be neglected when evaluating the environmental MP contamination [26, 27], especially when considering the increased potential toxicity of small MP [28]. Due to their miniscule size and high abundance, manual analysis of S-MP is not feasible. Thus, rapid, automated and highly accurate analytical methods are required that allow for a high-throughput rate of samples. Well-established analytical methods include pyrolysis–gas chromatography (py-GC) coupled with mass spectrometry (MS) [29–31] or thermal extraction desorption (TED) GC-MS [32, 33] which, however, only give information on polymer composition and polymer masses in a sample. In contrast, spectroscopic methods such as focal plane array (FPA) based micro-Fourier-transform infrared (µFTIR) imaging [24, 25, 34] and µRaman mapping [25, 35, 36] have proven to be highly efficient methods for MP analysis of sample filters, yielding information not only on polymer type but also on item count, shape and size distributions.

This study focuses on FPA-µFTIR analysis of S-MP derived from environmental samples as this technique facilitates chemical imaging by simultaneously recording thousands of spectra within one measurement in a reasonable timeframe, which makes it a powerful tool in MP analysis of whole sample filters [34]. Previously, the IR-spectra of each item on the filter containing the purified sample were compared manually to reference spectra, which is prone to human bias. Due to the prevalence of non-plastic residues post purification and the potentially high abundance of S-MP in environmental samples, this, however, is extremely time-consuming and therefore not practical, especially for monitoring studies where a high number of samples need to be analysed. To facilitate a rapid analysis without manual screening, a broad range of algorithms have been developed to automate the process of spectroscopic MP data analysis. As explained by Hufnagl et al. [37], these can be categorised into model-based [38–44] and instance-based [2, 45–52] machine learning approaches. Model-based approaches are based on a statistical model from spectroscopic reference data which is then applied to unknown spectra. These are then assigned to predefined classes which may include anything from polymer types to matrix components such as sediment, plant or animal debris. Instance-based approaches, in contrast, directly apply the reference data (i.e. the “instance”) to identify unknown spectra based on similarity measures. Here, hit quality indices (HQIs) are computed, e.g. by measures such as the Pearson correlation coefficient [37, 48]. The advantage of this approach is that the spectroscopic reference data can easily be enhanced or adapted, e.g. by adding relevant spectra to the existing library [53]. For model-based learning, however, a high degree of expert knowledge is required which make application-specific changes more difficult. On the other hand, it has the advantage of much shorter analysis time which allows for a higher analytical throughput [37].

In the field of MP research, it is well recognised that the comparison of results from different studies is often hampered by the lack of standardisation concerning sampling methods, sample processing and analytical methods. Discrepancies can of course also arise from different automated data evaluation algorithms at the end of the analytical pipeline. Thus, as a first step towards harmonisation in this regard, the aim of our study is a comprehensive comparison of the output of two frequently used and well-established FPA-µFTIR data analysis algorithms: (1) siMPle (systematic identification of MicroPlastics in the environment) (which is freely accessible on www.simple-plastics.eu) [48] in combination with the image analysis tool MPAPP (MicroPlastic Automated Particle/fibre analysis Pipeline), with its script having been published by Primpke et al. [54] (available for download as executable here: https://drive.google.com/drive/folders/1fWIGp7MgJZJcy7NWI5Vri0eUYJw0Qvrz?usp=share_link), and (2) the Bayreuth Particle Finder (BPF), which is based on the methodology presented in Hufnagl et al. [42] and is an integrated module in the Epina ImageLab Engine (www.imagelab.at). BPF is the preliminary version of the Purency Microplastic Finder developed by the Purency GmbH, which is commercially available (https://www.purency.ai/microplastics-finder). The main difference between both approaches is that siMPle is an instance-based machine learning approach and relies on a dual database search using two different similarity measures. HQIs are computed through Pearson correlation [48]. BPF on the other hand is a model-based machine learning approach. Here, a combination of spectral descriptors and random decision forest (RDF) classifiers is applied [42]. Both pipelines allow for the analysis of whole filters, avoiding the bias which would occur during extrapolation of results obtained from randomly preselected subareas of a filter [55]. Furthermore, they have been applied frequently in a multitude of different studies, analysing MP from various environmental matrices. BPF has, for instance, been applied in different studies focusing on MP contamination in freshwater environments, such as Frei et al. [56] and Schrank et al. [57]. It has further been applied in studies focusing on the analysis of airborne MP [6], MP in soil [58] but also MP in animal tissue [59]. Over the course of the years, the approach based on RDF classifiers after Hufnagl et al. [42] has undergone different development stages to improve on certain aspects of the classification, resulting in the latest version which was released in 2021 [37] and applied in Dong et al. [60]. For the present study, the version released in 2019 was chosen, as it had been applied in the above-mentioned previous studies. The analysis software siMPle also finds a broad range of applications, e.g. in the analysis of drinking water [61], wastewater [62] or marine waters [63]. In combination with MPAPP or its precedent version APA (automated particle analysis, after Primpke et al. [2]), siMPle has been applied in recent studies on river surface water [64, 65], wastewater effluents [53, 66] and deep sea sediments [67].

Within this study, we have analysed two sample sets containing ten measurement files each, which were both analysed by the algorithms siMPle/MPAPP and BPF. The resulting data output was compared with respect to (a) MP abundance, (b) polymer composition and (c) size distribution. Hereby, the present work can enhance the understanding of similarities and differences of MP analysis pipelines, with the ultimate goal of forming a basis for a better comparability of MP datasets from different studies. It is to be noted that the two sample sets used for the comparison of the two aforementioned analysis algorithms are based on environmental samples. Thus, the actual amount of MP in the samples is unknown, but the comparison of the output data is nevertheless valid. We chose to work with environmental samples in order to demonstrate the complexity of the analysis of these samples, representing all environmentally relevant polymer types, shapes and sizes as well as environmentally aged MP, potentially containing matrix residue and remains of biofilm. As mentioned above, S-MP is much more abundant in the environment and poses a higher ecotoxicological risk. Thus, the importance of evaluating methods suitable for the analysis of S-MP is enhanced and our study aims at shedding light on this matter.

## Materials and methods

In order to compare the output of the two analysis pipelines, two MP sample sets (size range: 11–500 µm) — sample set A containing ten riverine samples and sample set B containing ten estuarine samples — were analysed with the BPF [42] and in parallel with the MP analysis tools siMPle and MPAPP [48, 54]. The measurement files re-analysed in the present work had been generated in the framework of the joint project PLAWES (Microplastic Contamination in the Weser-Wadden Sea-National Park Model System: an Ecosystem-Wide Approach), which aimed at a comprehensive assessment of MP in the river system Weser-Wadden Sea. Herein, sample set A contains water samples from the Upper and Middle Weser and is subject of a study by Moses et al. (unpublished data). Sample set B contains water samples collected in the Lower/Outer Weser and Jade Bay and was initially analysed by Roscher et al. [64] (Tab. S1).

After enzymatic-oxidative sample purification based on the protocol presented in Löder et al. [68], all samples had been filtered onto Anodisc™ filters (aluminium oxide, pore size: 0.2 µm, diameter: 25 mm, Whatman, UK). Measurements using µFTIR imaging (Bruker Hyperion 3000, Bruker Optik GmbH, Germany) were performed with a 64 × 64 FPA detector in transmission mode with a 3.5 × IR lens in the wavenumber range 3600 − 1250 cm^−1^ (spectral resolution: 8 cm^−1^) using 32 co-added scans (background on pure filter: 32 and 64 scans, respectively), resulting in a pixel resolution of 11 µm. Data were saved as OPUS-measurement files (operating software OPUS 7.5, Bruker Optik GmbH, Germany). Details on the subsequent data analysis through siMPle/MPAPP and BPF are provided in the following sections.

### siMPle/MPAPP

OPUS-measurement files of sample set A and B were first processed with the software OPUS 7.5 (Bruker Optik GmbH, Germany), in order to transfer spectral data into the file format.dx in preparation to the following analysis steps [48]. In siMPle (version 1.1.β), the.dx files were converted into the .spe format, allowing for the subsequent automated comparison to our in-house polymer database [47]. Within this process, each spectrum is compared twice with the database (siMPle_database_Version 1.0.1), first using the untreated spectra and a second time using the 1st derivatives for spectral correlation calculation. Only if both processes determine the spectrum of the same polymer type, it is labelled as correctly identified for later image analysis, following the approach from Primpke et al. [2]. The data processing in siMPle was followed by the final image analysis via MPAPP. Here, the determined image containing the *x*,*y* coordinates on the filter, the combined hit quality and assigned polymer type are first analysed against polymer-specific quality control threshold values [69] for each polymer type. This is followed by a majority voting filter analysis and a series of image analysis tools to separate fibre like items from particles (see Primpke et al. [54] for the exact details of the procedure). Sample set B underwent an additional processing step, where the pixels assigned to the polypropylene (PP) support ring of the Anodisc filters containing the sample were removed in OriginPro.2017G (OriginLab Corporation, USA) (size of measurement field: 20 × 20–22 × 22 FPA tiles). This step was not necessary for sample set A, as a smaller measurement field had been applied (17 × 17 FPA tiles), which did not cover any border area of the filter. The final analysis using MPAPP provided information on numbers, sizes and polymer composition of particle and fibre-like MP items [54].

### Bayreuth Particle Finder (BPF)

Both datasets analysed with BPF were also first processed with the software OPUS 7.5 (Bruker Optik GmbH, Germany), in order to transfer spectral data into the file format.hdr while the optical image was extracted as .jpeg file. The .hdr file containing the spectral data and the infrared image of the analysed filter were then imported to the program ImageLab (Epina GmbH) [42]. The optical image was then aligned with the infrared image and carefully adjusted manually. After calibration with measurement parameters from the OPUS measurement file, the BPF algorithm was applied to the measurement data. The database used to train the machine learning model contained 22 polymer types (for details see Table 1). In the classification step, each pixel is classified by the machine learning model resulting in the assignment to either a polymer type or a class describing the matrix (“non-plastic”) or the filter surface (“background”). MP items are detected as neighbouring pixels of the same polymer class. The results are presented in a list containing their properties such as the size (longest and shortest dimension), polymer type, position on the filter etc. All material identified and categorised as “non-plastic” and “background” were deselected. The spectra of the remaining items were checked twice by experts. This conservative step is optional but was included in our routine for QA/QC and more reliable results. In Hufnagl et al. [37], cases are discussed, where expert intervention can significantly improve questionable results, e.g. coming from total absorption and overlapping MP items. For this, the spectra of items identified as plastic were compared using a built in reference database. Furthermore, the size of the identified polymer was verified with the optical and infrared image and if necessary adapted using the editing tools. This allowed to correctly assign items that are partially covered with organic material post-purification that may mistakenly be registered as smaller items. Furthermore, due to the round cross section of synthetic fibres and resulting distorted IR spectra, oftentimes only individual pixels of fibres are identified as plastic. Therefore, the entire length and width of the fibres were audited to allow an exact size measurement. The generated output file delivers information on polymer type, shape, length and width of the identified items.

**Table 1:** Harmonised polymer types compared in this study, as well as excluded ones, which were only present in one of the analysis pipelines. Abbreviations: *A*: acrylates; *CA*: cellulose acetate; *CMC:* chemically modified cellulose; *EVA and EVAc*: ethylene vinyl acetate; *PA*: polyamide; *PC*: polycarbonate; *PP*: polypropylene; *POM*: polyoxymethylene; *PEEK*: polyether ether ketone; *PVC*: polyvinylchloride; *PUR/PU*: polyurethane; *PMMA*: polymethyl methacrylate; *PS*: polystyrene; *ABS*: acrylonitrile butadiene styrene; *PEST*: polyester; *PET*: polyethylene terephthalate; *PBT*: polybutylene terephthalate; *PSU*: polysulfone; *PLA*: polylactic acid; *PLA-PBAT*: polylactic acid/poly(butylene adipate-co-terephthalate) blend; *PE*: polyethylene, *V*: varnish

Polymer type	BPF	siMPle/MPAPP
Harmonised polymer types/clusters		
CA	CA	CMC
EVA	EVAc	EVA
PA	PA	PA
PC	PC	PC
PP	PP	PP
POM	POM	POM
PEEK	PEEK	PEEK
PVC	PVC	PVC
A/PUR/V	PU, PMMA	A/PUR/V
PS	PS, ABS	PS
PEST	PET, PBT	PEST
PSU	PPSU, PSU	PSU
PLA	PLA, PLA-PBAT	PLA
PE	PE	PE, PE-oxidised

Polymer types only present in one pipeline (excluded from analysis)		
	EVOH	-
	PAN	-
	SIL	-
	-	PE-chlorinated
	-	Nitrile rubber
	-	Polyimide
	-	Polychloroprene
	-	Polyisoprene-chlorinated
	-	PCL
	-	Polybutadiene
	-	Acrylonitrile-butadiene
	-	Rubber type 1
	-	Rubber type 2
	-	Rubber type 3

### Data evaluation

In order to compare the datasets generated by the siMPle/MPAPP and BPF analysis, MP polymer data were harmonised. For this, comparable polymer types (e.g. containing same functional groups, such as terephthalates or styrenes) were identified, and grouped into clusters whenever necessary (Table 1). For instance, the terephthalates, polybutylene terephthalate (PBT) and polyethylene terephthalate (PET), are identified separately in the BPF. The siMPle output data, however, shows results on the cluster polyester (PEST), which includes both aforementioned terephthalates [47]. This resulted in the harmonised polymer cluster PEST, which for the BPF output data includes PET and PBT. In contrast, some polymer types were only present in one of the analysis pipelines (e.g. polyacrylonitrile (PAN), polyimide or polychloroprene; (Table 1)) and were therefore excluded from the comparison.

## Results

### Exclusion of further polymer clusters from analysis

**Fig. 1:**
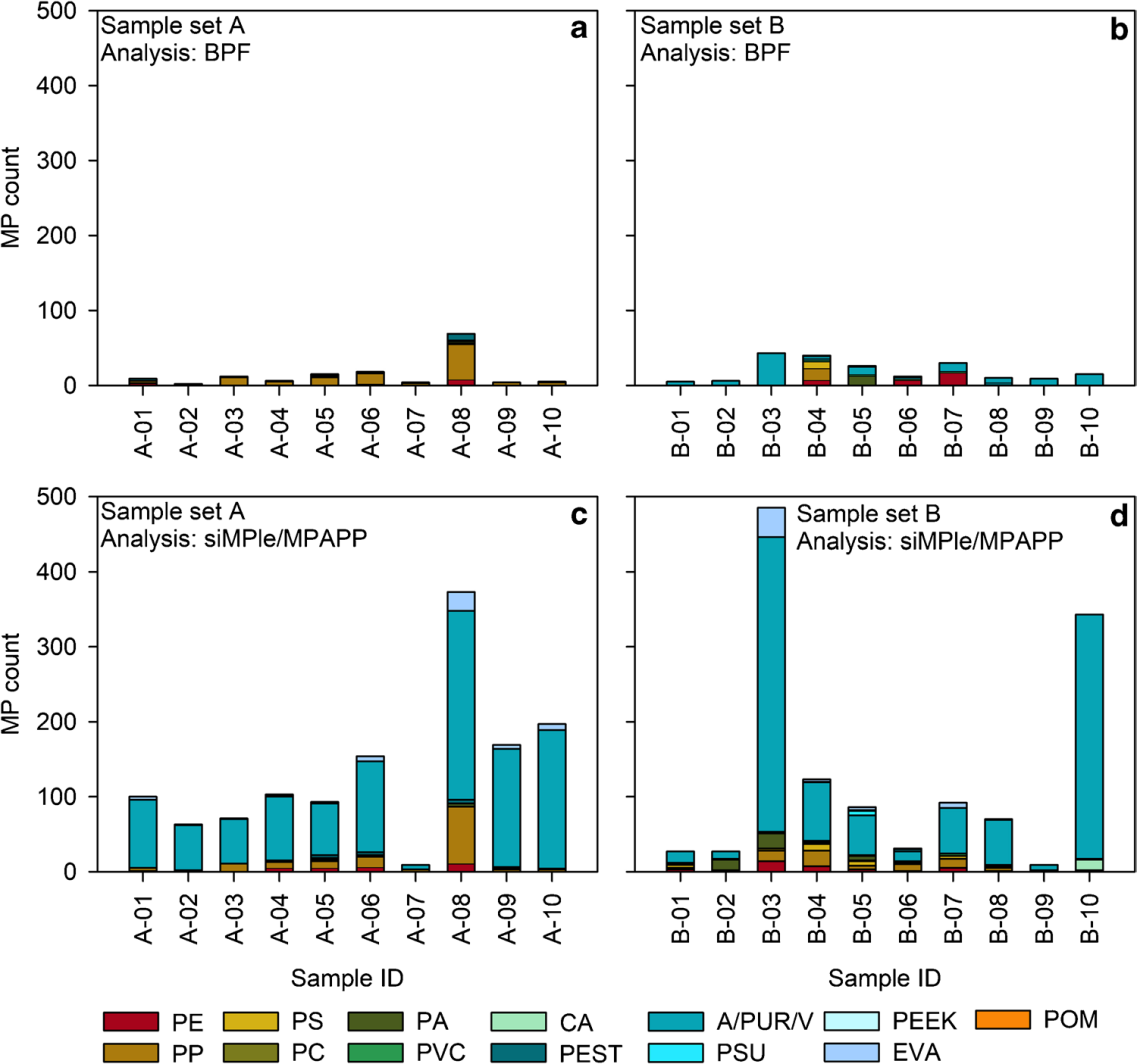
Data output after BPF (**a**, **b**) and siMPle/MPAPP (**c**, **d**) analyses of sample set A and B before exclusion of EVA, CA and A/PUR/V (cf. the “Exclusion of further polymer clusters from analysis” section). For abbreviations refer to Table 1

When comparing the data output of BPF and siMPle/MPAPP, we noticed that the polymer type ethylene vinyl acetate (EVA) was relatively prominent in the siMPle/MPAPP data (*n* = 55 in both sample set A and B, respectively), whereas no assignments to EVA were reported after BPF analysis (see Tables S2 and S4). The same was true for cellulose acetate (CA), with no detections after BPF analysis, but 14 assignments (sample set B) after siMPle/MPAPP analysis. With respect to EVA, 10 exemplary spectra assigned to EVA by siMPle were re-inspected together and rejected in agreement of both operators, due to poor hit quality. Furthermore, strong discrepancies in the data output were also recorded for the acrylates/polyurethanes/varnish (A/PUR/V) cluster, with much higher counts after siMPle/MPAPP analysis (*n* = 1086 and *n* = 1016 in sample set A and B, respectively) compared to after BPF analysis (*n* = 4 and *n* = 108 in sample set A and B, respectively) (Fig. 1). Thus, the clusters EVA, CA, and A/PUR/V were excluded from further analysis. Reasons for the differences will be discussed in detail in the “Effects of differences in the general methodological approach” section.

### MP abundance, polymer composition and size distribution

**Fig. 2:**
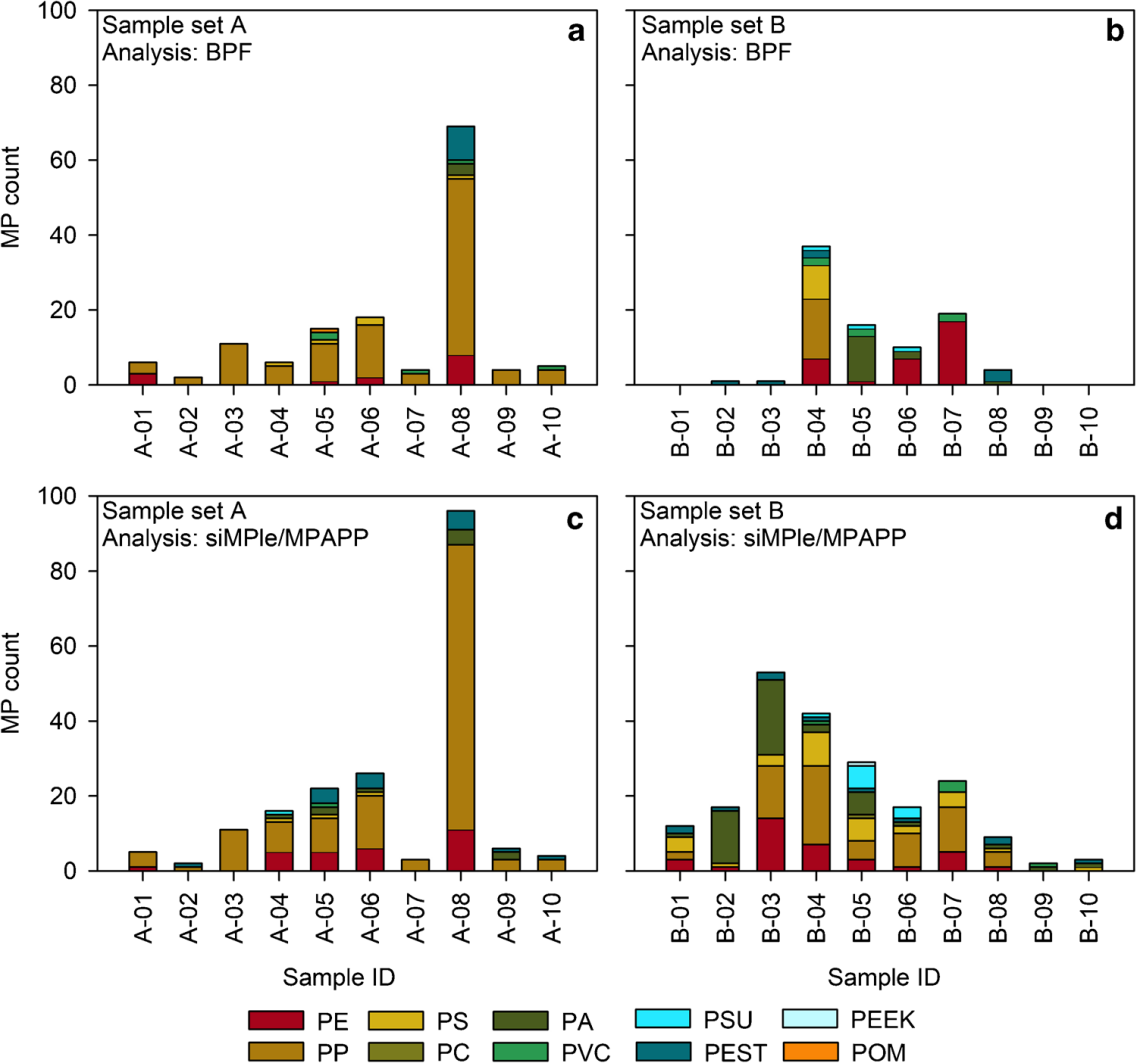
Data output after BPF (**a**, **b**) and siMPle/MPAPP (**b**, **c**) analyses of sample set A and B, under exclusion of EVA, CA and A/PUR/V (cf. the “Exclusion of further polymer clusters from analysis” section). For abbreviations, refer to Table 1

Comparison of the analysis output of siMPle/MPAPP and BPF after exclusion of previously mentioned polymer types/clusters (EVA, CA and A/PUR/V) revealed similar results for sample set A (Fig. 2a, c), with a difference in polymer count detected by both pipelines being on average ∆n ~ 6 (mean ± standard deviation (SD) = 6 ± 8), with ∆n ranging from 0 for A-02 and A-03 to 27 for A-08. Samples with a difference of ∆n > 6 were A-04 (∆n = 10), A-05 (∆n = 7), A-06 (∆n = 8) and A-08 (∆n = 27). Within both pipelines, PP and PE were identified as dominant polymer types and maximum item counts were recorded for sample A-08 (Fig. 2a, c). Furthermore, in all remaining samples (except for sample A-04), a similar trend was observed.

**Fig. 3:**
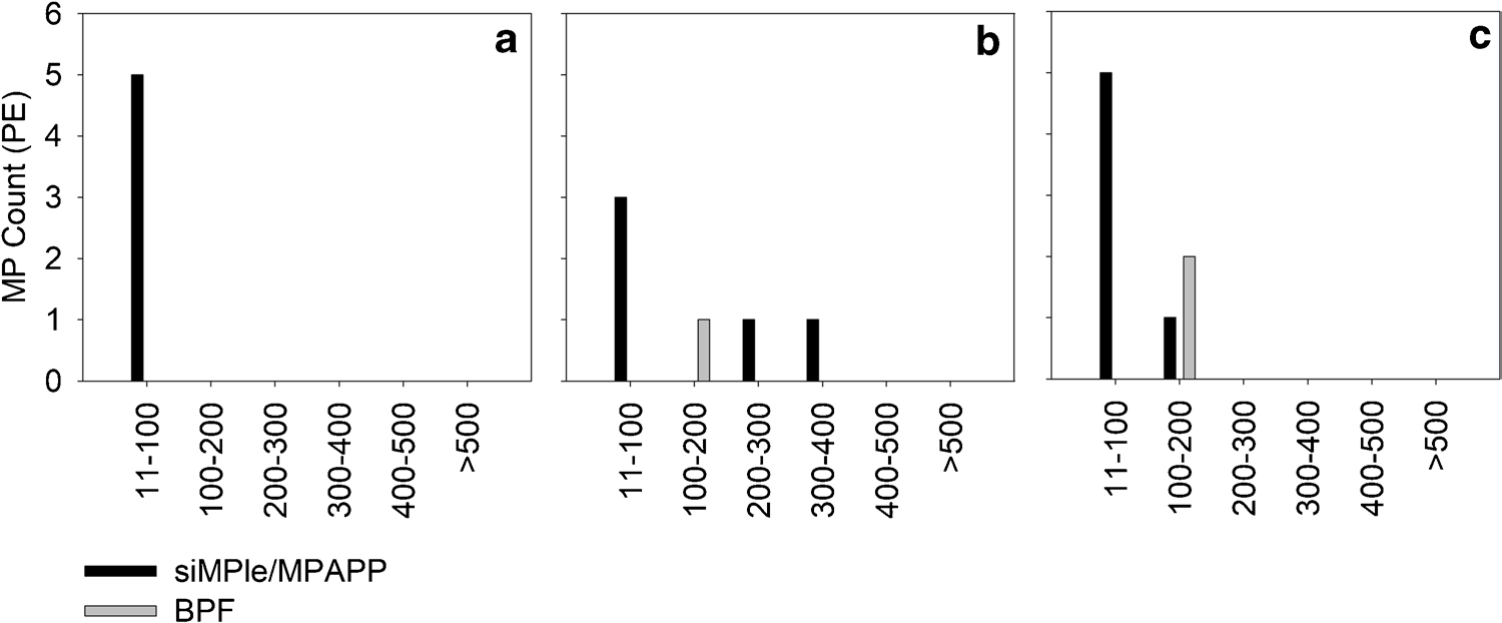
Assignments to PE in samples A-04 (**a**), A-05 (**b**) and A-06 (**c**) by siMPle/MPAPP (black) and BPF (grey), shown for different size classes. A share of 85% of the assignments to the 11–100 µm size class refers to MPs  <25 µm

Generally, in the riverine sample set A, the siMPle/MPAPP pipeline detected more PA (present in five samples, 1–4 items per sample) and PEST (present in six samples, 1–5 items per sample) than the BPF pipeline (PA present in one sample containing 3 items; PEST present in one sample containing 9 items). Slight differences were also recorded with regard to PE: In samples A-04, A-05 and A-06, siMPle/MPAPP detected more PE than BPF (Fig. 2, ∆n = 5, 4 and 4, respectively). On the other hand side, BPF detected more PVC (four samples, 1–2 items per sample) than siMPle (one sample containing 1 item). Details can be found in Table S2 and Table S3. The differences in PE in the aforementioned samples were especially recorded for items  <100 µm (Fig. 3), and herein the majority of MP detected by siMPle/MPAPP had a size  <25 µm (85%). A similar observation was made for items identified as PA (70% < 25 µm, *n* = 7) and PEST (100% < 25 µm, *n* = 16) by siMPle/MPAPP within sample set A, which were rather present in larger size classes based on BPF results.

With respect to the estuarine sample set B, a decreasing trend in the MP counts was recorded from sample B-04 to B-10 with both analysis pipelines (Fig. 2b, d). The other samples, however, showed a stronger variation between pipelines: samples B-01, B-02 and B-03 showed 0–1 MP item after BPF analysis, whereas siMPle detected  >10 MP (B-01 and B-02), and  >50 MP (B-03). Concerning polymer compositions, sample B-04 showed a high degree of similarity after BPF and siMPle/MPAPP analysis, with PE, PP and PS being predominant polymer types. The results of the remaining samples, however, showed higher discrepancies. For samples B-06 and B-07, e.g. BPF detected more PE, whereas after siMPle analysis, PP was predominant. Details can be found in Table S4 and Table S5. Here too, with regard to PE, the differences were mainly driven by the smallest size class 11–25 µm, with *n* = 6 (sample B-06) and *n* = 13 (sample B-07) MP items detected by BPF, while 0 and 3, respectively, were detected by siMPle/MPAPP. This contrasted with the findings for sample set A, where more small PE items were detected by siMPle/MPAPP (Fig. 3).

**Fig. 4:**
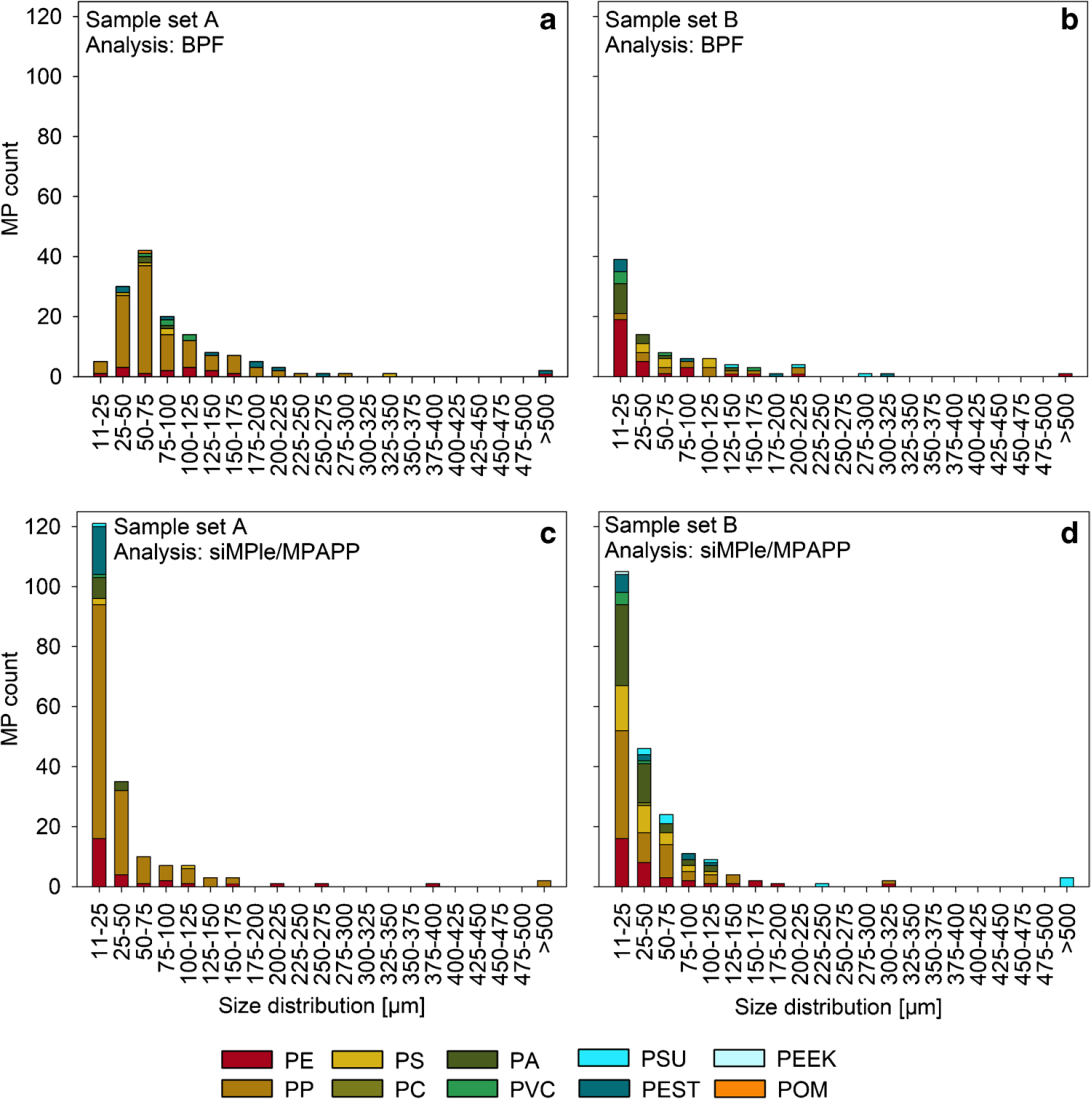
Overall size distributions (length of detected MP in µm) recorded for sample set A and B through BPF (**a**, **b**) and siMPle/MPAPP (**c**, **d**) analysis. It is to be noted that all size bins cover 25 µm, except for the smallest category. The latter starts at 11 µm which is equivalent to the size of one FPA detector pixel

The overall size distribution presented in Fig. 4 confirms the impression of size-related discrepancies, which were already stated above for PE, PEST and PA. For sample set A, few counts in small size classes were recorded in the BPF results, and most MP were assigned to the 50–75 µm size range (Fig. 4a). In contrast, siMPle results were clearly dominated by MP items in the size class 11–25 µm (Fig. 4c), with PP, PEST and PE being dominant. With regard to sample set B, both analyses showed highest counts in the smallest size class (Fig. 4b, d). However, especially in this size class, polymer compositions differed strongly with PE being dominant in the BPF results, whereas PP but also PA were dominant in siMPle/MPAPP results.

## Discussion

### Effects of MP size on spectral quality and automated analysis

This study provides a detailed comparison of two MP analysis pipelines, i.e. siMPle/MPAPP and BPF. It is to be noted that this comparison was conducted with environmental samples, containing an unknown amount and composition of MP items. Thus, it remains unclear which results of the aforementioned analysis pipelines are closer to the actual MP occurrence. However, this approach was chosen to shed some light on the complexity of the analysis of environmental samples. Working with internal standards or spiked samples has multiple limitations in terms of representativeness due to the limited availability of commercially available reference material in regard to polymer types, shapes and sizes. Additionally, the preparation of spiked MP samples is challenging, especially using S-MP < 100 µm [70]. As this size range, however, appears to be the most abundant in environmental samples and poses an increased ecotoxicological risk, it is especially important to represent this size class. The aim of our study, however, was to demonstrate the complexity of the analysis of environmental samples in its full spectrum, containing all relevant polymer types, shapes and sizes, as well as the interplay of matrix residues with aged microplastic being the real challenge for both algorithms. Furthermore, a well-established approach in other domains of chemometrics for comparing algorithms is the use of expert-annotated training data [71, 72]. However, within the considered size range, experimental difficulties (e.g. the handling of small virgin MP items) make it very challenging to ensure a correct assignment of the ground truth of spectra, which is why, as a first step, this study was limited to a relative comparison of results. Nevertheless, our study highlights the similarities and differences in results obtained with both tools which are essential for further efforts towards method optimisation and harmonisation.

Despite of the use of state-of-the-art methods, our results, however, also underline the uncertainties in MP analysis concerning polymer identification, especially at the lower end of the detection limit in terms of size. Depending on morphology and thickness of MPs in a size range below 50 µm, IR-spectra can be influenced by effects such as diffraction and Mie scattering [42], in tandem with low intensity of the original polymer signal. Thus, the interplay of these phenomena potentially results in low quality spectra and low signal-to-noise ratios. Due to a more conservative, time-intensive approach with BPF, possibly such low-quality polymer spectra were manually rejected. While for sample set A between 17 and 50% (mean ± SD = 37% ± 9%) of all items identified as MP were accepted after manual re-inspecting the data, only between 0 and 39% (mean ± SD = 13% ± 15%) of all MP hits were accepted for sample set B. For siMPle/MPAPP, such a process was performed former to this study in previous work on surface water samples collected in the North Sea [69], setting the minimum threshold to be reached per polymer type. Nevertheless, during application of siMPle/MPAPP, a fraction of such low quality spectra may have been included automatically. Although the problem of classification of MPs with low quality spectra at the lower end of the detection limit of IR spectroscopy has been demonstrated in our case on the example of two automated analysis algorithms, it also holds true for other automated classification solutions or pure manual classification. The severity of this phenomenon may differ between studies as it also depends on the specifications of the respective µFTIR system, filter type etc. used for MP sample measurement.

###  Potential reasons for differences in MP abundance, polymer composition and size distributions

 Our results showed a high similarity between MP counts and polymer composition especially with respect to sample set A (average difference: ∆n ~ 6, maximum difference: A-08, ∆n = 27), where PP and PE were recorded as most abundant polymer types (Fig. 2a, c). Slight differences in polymer compositions, however, were observed: more PE and PEST were detected by siMPle/MPAPP than with BPF (samples A-04, A-05, and A-06). As stated above, these differences were mostly driven by the spectral features occurring in the smallest size classes.

With regard to sample set B, most samples showed similar MP counts after BPF and siMPle/MPAPP analysis (average difference: ∆n ~ 12). Additionally, sample B-04 showed strikingly high similarities also with regard to polymer compositions (Fig. 2b, d). Discrepancies in MP counts, however, were observed in samples B-01 to B-03 (maximum difference: B-03, ∆n  >50). For B-01 and B-02, this is possibly due to a matrix effect: these samples were rich in very fine sediments that could not be effectively eliminated during sample purification, which was underlined by high content of suspended particulate matter (SPM) (details in Table S1) recorded during sampling in the semi-enclosed Jade Bay (German North Sea) [64]. These characteristics, potentially resulting in matrix interferences, were in contrast to the other samples from sample set B and might have negatively affected the automated polymer identification by either under- or overestimation by the respective analysis pipelines. This problem can only be solved in future by further adjustments in the extraction and purification methodology. Sample B-03, however, did not show any obvious specific matrix-related characteristics which would explain the observed differences between both pipelines. Concerning the remaining samples of sample set B (B-04 to B-10), a general trend of decreasing MP counts was observed for both pipelines. However, differences in polymer compositions became evident especially in B-06 and B-07, where BPF detected more PE, whereas siMPle/MPAPP detected more PP. Randomly selected PP spectra detected through siMPle analysis were visually checked (exemplary spectra shown in Fig. S1) and accepted, as characteristic bands were present (i.e. stretching vibrations of CH_3_ and CH_2_ between  ~2830 and 2950 cm^−1^, and bending vibrations of mainly CH_3_ at  ~1450 and  ~1370 cm^−1^). However, existing noise in the spectra might have resulted in manual rejection during the quality check after BPF analysis, while it was considered valid due to double spectral confirmation within the siMPle process, as described in Primpke et al. [48]. The differences in PE counts, however, could not be explained in the framework of this study. Finally, our analysis showed that for the polymer clusters considered, BPF and siMPle/MPAPP generally are in accordance with regard to MP counts and polymer compositions, with exceptions likely caused by residues of complex sample matrixes post sample purification on the sample filters and discrepancies regarding the detection of small MP items  <25 µm.

Both pipelines found an overall dominance of MP in the smaller size classes. However, especially for sample set A, the overall size distribution differed between BPF and siMPle/MPAPP results, with the latter showing much higher counts in the smallest size class. Next to low quality spectra resulting from diffraction, Mie scattering and low signal intensity that may have led to an under- (BPF) or overestimation (siMPle/MPAPP) in the smallest size classes by the respective methods as described above, these discrepancies are potentially also due to different assessment approaches with regard to size classification in both algorithms. After the application of BPF, a manual QA/QC was performed for each classified MP item, and whenever necessary adjacent pixels were combined retrospectively if they clearly belong to the same item. During siMPle/MPAPP analysis, a closing step is implemented, where neighboured pixels are combined automatically [2, 54]. Thus, if the surface of an item is not uniform, potentially due to organic residues, i.e. the closing step may not be successful. This may lead to the detection of multiple counts in smallest size classes instead of the detection of one large item, which could be the reason for the observed differences in size classification. Nevertheless, a general high occurrence of small MP items was confirmed by Primpke et al. [73] using QCL-IR measurements and Cabernard et al. [74] with both µFTIR and Raman, with the latter showing even higher counts in the exact same sample. Thus it remains unclear, to what extent the aforementioned effect is relevant for the differences in size distributions observed in the present study.

Consequently, the observed differences between BPF and siMPle/MPAPP concerning size distributions underline the necessity of further comparative studies and should be focus of future harmonisation efforts with the final goal of a reliable assessment of environmental MP concentrations in all detectable size classes.

### Effects of differences in the general methodological approach

To our knowledge, this is the first study to compare a model-based to an instance-based algorithm with respect to resulting MP abundances, polymer compositions and sizes, by applying both analysis pipelines on the data of the same two MP sample sets. For an accurate comparison of BPF and siMPle/MPAPP, a restructuring of the data output was performed, including the harmonisation of polymer types and the exclusion of those which are not targeted in both pipelines (e.g. silicone, which is only addressed by BPF, or polyimide, which is only included in siMPle/MPAPP; Table 1). This data handling step resulted in the data output presented in Fig. 1, showing that especially A/PUR/V was much more frequently detected by siMPle/MPAPP. Both, the PUR and PMMA class of the BPF approach was trained using only PUR and PMMA spectra. In siMPle/MPAPP, the individual spectra of different polymers were assigned by a label to polymer type clusters of similar substances [47] including other types of acrylate substances for A/PUR/V. While the former approach tries to mimic the real structure of the underlying polymer data by deriving a statistical model, the latter uses a combination of hierarchal cluster analysis and expert knowledge to generate generalised polymer type clusters separable in the available spectral range. The harmonisation step should thus be observed critically, as different design philosophies, with very different mathematical characteristics are compared. This potentially leads to a broader coverage of polymers by siMPle/MPAPP, while the BPF database allows that certain polymer types such as PUR and PMMA are differentiated.

Interestingly, BPF detected items falling into the A/PUR/V cluster especially in sample set B (Weser-Wadden Sea transitional zone), but almost none in sample set A (Upper and Middle Weser) (Fig. 1). Sample set B originates from sampling stations situated in an area with relatively high shipping activity, which represents a potential source for varnish-like items [64]. In this study, also L-MP items with a varnish-like morphology were recorded in this area. Sample set A, however, stems from an area less influenced by shipping activity in the middle and upper part of the Weser River, which is in accordance with very few (*n* = 4) validated assignments to the cluster A/PUR/V by BPF, but contrasts with the extremely high abundances after siMPle/MPAPP analysis (*n* = 1086). Beside the possibility of potential underestimation of this polymer cluster by BPF due to conservative manual rejection of low-quality spectra, another explanation could be an overestimation by siMPle/MPAPP, e.g. due to the missing awareness of specific natural materials causing systematic false positive assignments. This cluster was addressed with an extensive QA/QC procedure in a previous study on MP in WWTP effluents. It was found that this polymer cluster is strongly affected by a spectral interference with plant cuticles in sample matrixes present in high amounts of residual biological material [53] resulting in false positives with a high spectral match. Hence, in sample set A, an overestimation in samples with a high organic load by siMPle/MPAPP in the A/PUR/V cluster cannot be ruled out. Also, the polymer cluster EVA showed significant discrepancies, with relatively high abundances after siMPle/MPAPP analysis (sample set A: *n* = 55, sample set B: *n* = 55), and no counts after BPF analysis. As stated in the “Exclusion of further polymer clusters from analysis” section, respective siMPle-spectra were rejected after visual re-inspection by both operators as false positives. Also here, the manual inspection of the analysis results as routinely implemented after BPF analysis leads to exclusion and more conservative results with regard to this polymer type. Especially the lack of the ethylene signal at approx. 1370 cm^−1^ in the sample spectra (Fig. S2) led to rejection of most spectra assigned to EVA. Moreover, similar to the siMPle cluster A/PUR/V, also EVA was affected by matrix interferences in the study by Roscher et al. [53], further hinting towards the assumption that respective counts in the present study might also be due to false positive identification. Indeed, the two samples with highest EVA counts (A-08: *n* = 25; B-03: *n* = 39) showed high amounts of potentially biogenic material on Anodisc™ filters (Fig. S3) in comparison to other samples. These observations show that despite the high benefit of automated or semi-automated analysis pipelines, expert knowledge and manual QA/QC processes are highly necessary in order to allow for solid and unbiased datasets, as previously stated by Song et al. [75]. In siMPle/MPAPP, a QA/QC on the individual spectral level is implemented on a regular basis (see Lorenz et al. [69] and Primpke et al. [48]), whereas in BPF, the manual check of MP assignments was performed routinely for each dataset. Although the latter may provide a high certainty, it can also be time consuming, depending on the amount of potential MPs detected. For example, as stated above, in sample set A 50–73% (mean ± SD = 63% ± 9%) and in sample set B 61–100% (mean ± SD = 87% ± 15%) of all items identified as MP by the BPF algorithm were rejected after manual reinspection of the spectra. Here, especially samples with a high content of residual matter post-purification (such as fine sand or non-digestible matter such as plant pollen) appear to be critical. Due to an increased amount of material remaining on the filter, the time required for manual re-inspection of all spectra assigned to MP increases. These observations underline the great importance of an effective purification approach to produce final MP samples with as little sample matrix as possible present [68].

Due to the IR-transparency features of the filter material (aluminium oxide, Anodisc) used as substrate for FTIR imaging in this study, only the wavenumber range 3600 − 1250 cm^−1^ could be measured, where synthetic and biogenic substances partly share similar bands and discrimination can be challenging [53]. Thus, the additional assessment of data in the spectral fingerprint range  <1250 cm^−1^ should be further pursued for better distinguishing materials. In the case of µFTIR imaging in transmission mode, which results in the highest spectral quality [34], this is only possible if the used filter material aluminium oxide is replaced by material like silicon that is also IR transparent in the fingerprint region [76]. This filter substitute could help to improve classification especially for acrylate- and PUR-based polymers as well as EVA which would enhance the comparability of results as well as the general detection success and reliability of data.

### Future implications

By comparing two currently well-established and frequently applied MP analysis tools, this study can act as a basis for future harmonisation and standardisation efforts in MP analysis. In general, BPF and siMPle/MPAPP showed similar results, with some discrepancies likely caused by matrix effects, and others explainable by the chemical characteristics of certain polymers which could be improved by a broader measurement range including the fingerprint region. On the whole, both pipelines are rapid and generate a detailed data output and therefore show great potential for a broad application in MP assessments. This study further underlines the importance of QA/QC, e.g. implemented by manual counter-checking by experts, in order to allow for the generation of high-quality datasets and underlines the importance of purification approaches that reduce the present sample matrix effectively. Additionally, our study also shows that all studies on MP contamination should be interpreted with caution, especially with respect to smaller size classes, since it remains unclear for all currently applied methods how correct the generated results are with respect to the actual occurrence of MP in the environment. Keeping this in mind, as a final consequence, we have to admit that even by the use of state-of-the-art methodology, the determination of the real environmental MP number is still a challenge which needs to be addressed by further research efforts. In order to have a clearer picture of how close the obtained results are to the actual numbers, it may perhaps be beneficial to work with spiked samples — although one has to be aware of the limitations in regard to available polymer types, shapes and sizes. Nevertheless, through the current ongoing development and improvement of the here applied analysis tools, both usability and reliability are being enhanced, e.g. by adaptations of underlying reference databases (siMPle) or optimised follow-up versions (“Purency Microplastic Finder” derived from BPF). These improvements and further optimisations will lead to analysis tools that — in the best case — produce data with high reliability without additional manual re-evaluation efforts.

## Supplementary Information

Below is the link to the electronic supplementary material.

Supplementary file1 (PDF 581 KB)

## References

[1] Lebreton LCM, van der Zwet J, Damsteeg JW, Slat B, Andrady A, Reisser J (2017). River plastic emissions to the world’s oceans. Nat Commun.

[2] Primpke S, Lorenz C, Rascher-Friesenhausen R, Gerdts G (2017). An automated approach for microplastics analysis using focal plane array (FPA) FTIR microscopy and image analysis. Anal Methods.

[3] Cole M, Lindeque P, Halsband C, Galloway TS (2011). Microplastics as contaminants in the marine environment: a review. Mar Pollut Bull.

[4] Dris R, Gasperi J, Saad M, Mirande C, Tassin B (2016). Synthetic fibers in atmospheric fallout: a source of microplastics in the environment?. Mar Pollut Bull.

[5] Gasperi J, Wright SL, Dris R, Collard F, Mandin C, Guerrouache M (2018). Microplastics in air: are we breathing it in?. Curr Opin Environ Sci Health.

[6] Kernchen S, Löder MGJ, Fischer F, Fischer D, Moses SR, Georgi C, et al. Airborne microplastic concentrations and deposition across the Weser River catchment. Sci Total Environ. 2021;818:151812. 10.1016/j.scitotenv.2021.151812. 10.1016/j.scitotenv.2021.151812 34808158

[7] Rillig MC (2012). Microplastic in terrestrial ecosystems and the soil?. Environ Sci Technol.

[8] Rillig MC, Lehmann A (2020). Microplastic in terrestrial ecosystems. Science.

[9] Weithmann N, Möller JN, Löder MGJ, Piehl S, Laforsch C, Freitag R. Organic fertilizer as a vehicle for the entry of microplastic into the environment. Sci Adv. 2018;4:eaap8060. 10.1126/sciadv.aap8060. 10.1126/sciadv.aap8060 PMC5884690 29632891

[10] Peeken I, Primpke S, Beyer B, Gütermann J, Katlein C, Krumpen T (2018). Arctic sea ice is an important temporal sink and means of transport for microplastic. Nat Commun.

[11] Obbard R, Sadri S, Wong Y-Q, Khitun A, Baker I, Thompson R (2014). Global warming releases microplastic legacy frozen in Arctic Sea ice. Earth’s Future.

[12] Free CM, Jensen OP, Mason SA, Eriksen M, Williamson NJ, Boldgiv B (2014). High-levels of microplastic pollution in a large, remote, mountain lake. Mar Pollut Bull.

[13] Negrete Velasco AdJ, Rard L, Blois W, Lebrun D, Lebrun F, Pothe F, et al. Microplastic and fibre contamination in a remote mountain lake in Switzerland. Water. 2020;12(9):2410. 10.3390/w12092410.

[14] Scherer C, Weber A, Lambert S, Wagner M. Interactions of microplastics with freshwater biota. Freshwater microplastics: Springer, Cham; 2018. p. 153–80. 10.1007/978-3-319-61615-5_8.

[15] Sanchez W, Bender C, Porcher J-M (2014). Wild gudgeons (Gobio gobio) from French rivers are contaminated by microplastics: preliminary study and first evidence. Environ Res.

[16] Kögel T, Bjorøy Ø, Toto B, Bienfait AM, Sanden M. Micro- and nanoplastic toxicity on aquatic life: determining factors. Sci Total Environ. 2020;709:136050. 10.1016/j.scitotenv.2019.136050. 10.1016/j.scitotenv.2019.136050 31887526

[17] Carpenter EJ, Anderson SJ, Harvey GR, Miklas HP, Peck BB (1972). Polystyrene spherules in coastal waters. Science.

[18] Carpenter EJ, Smith KL (1972). Plastics on the Sargasso sea surface. Science.

[19] Norén F. Small plastic particles in coastal Swedish waters. KIMO Sweden, N-Research, Lysekil, Sweden. 2007.

[20] Shim WJ, Song YK, Hong SH, Jang M (2016). Identification and quantification of microplastics using Nile Red staining. Mar Pollut Bull.

[21] Cole M (2016). A novel method for preparing microplastic fibers. Sci Rep.

[22] Devriese LI, van der Meulen MD, Maes T, Bekaert K, Paul-Pont I, Frère L (2015). Microplastic contamination in brown shrimp (Crangon crangon, Linnaeus 1758) from coastal waters of the Southern North Sea and Channel area. Mar Pollut Bull.

[23] Löder MG, Gerdts G. Methodology used for the detection and identification of microplastics—a critical appraisal. Marine anthropogenic litter: Springer; 2015. p. 201–27. https://link.springer.com/chapter/10.1007/978-3-319-16510-3_8.

[24] Primpke S, Christiansen SH, Cowger W, De Frond H, Deshpande A, Fischer M (2020). Critical assessment of analytical methods for the harmonized and cost-efficient analysis of microplastics. Appl Spectrosc.

[25] Ivleva NP (2021). Chemical Analysis of microplastics and nanoplastics: challenges, advanced methods, and perspectives. Chem Rev.

[26] Huang D, Li X, Ouyang Z, Zhao X, Wu R, Zhang C, et al. The occurrence and abundance of microplastics in surface water and sediment of the West River downstream, in the south of China. Sci Total Environ. 2021;756:143857. 10.1016/j.scitotenv.2020.143857. 10.1016/j.scitotenv.2020.143857 33248769

[27] Hildebrandt L, Zimmermann T, Primpke S, Fischer D, Gerdts G, Pröfrock D. Comparison and uncertainty evaluation of two centrifugal separators for microplastic sampling. J Hazard Mater. 2021;414:125482. 10.1016/j.jhazmat.2021.125482. 10.1016/j.jhazmat.2021.125482 34030400

[28] Yong CQY, Valiyaveettil S, Tang BL (2020). Toxicity of microplastics and nanoplastics in mammalian systems. Int J Environ Res Public Health.

[29] Gerdts G, Thomas K, Herzke D, Haeckel M, Scholz-Böttcher B, Laforsch C, et al. Defining the baselines and standards for microplastics analyses in European waters (JPI-O BASEMAN). 2017. p. 120–2. 10.1016/B978-0-12-812271-6.00118-6.

[30] Fischer M, Scholz-Böttcher B (2019). Microplastics analysis in environmental samples – recent pyrolysis-gas chromatography-mass spectrometry method improvements to increase the reliability of mass related data. Anal Methods.

[31] Fischer M, Scholz-Böttcher BM (2017). Simultaneous Trace Identification and Quantification of Common Types of Microplastics in Environmental Samples by Pyrolysis-Gas Chromatography-Mass Spectrometry. Environ Sci Technol.

[32] Dümichen E, Barthel A-K, Braun U, Bannick CG, Brand K, Jekel M (2015). Analysis of polyethylene microplastics in environmental samples, using a thermal decomposition method. Water Res.

[33] Mansa R, Zou S. Thermogravimetric analysis of microplastics: a mini review. Environ Adv. 2021;5:100117. 10.1016/j.envadv.2021.100117.

[34] Löder MGJ, Kuczera M, Mintenig S, Lorenz C, Gerdts G (2015). Focal plane array detector-based micro-Fourier-transform infrared imaging for the analysis of microplastics in environmental samples. Environ Chem.

[35] Lenz R, Enders K, Stedmon CA, Mackenzie DMA, Nielsen TG (2015). A critical assessment of visual identification of marine microplastic using Raman spectroscopy for analysis improvement. Mar Pollut Bull.

[36] Käppler A, Fischer D, Oberbeckmann S, Schernewski G, Labrenz M, Eichhorn KJ (2016). Analysis of environmental microplastics by vibrational microspectroscopy: FTIR, Raman or both?. Anal Bioanal Chem.

[37] Hufnagl B, Stibi M, Martirosyan H, Wilczek U, Möller JN, Löder MGJ (2022). Computer-assisted analysis of microplastics in environmental samples based on μFTIR imaging in combination with machine learning. Environ Sci Technol Lett.

[38] Paul A, Wander L, Becker R, Goedecke C, Braun U (2019). High-throughput NIR spectroscopic (NIRS) detection of microplastics in soil. Environ Sci Pollut Res.

[39] Serranti S, Palmieri R, Bonifazi G, Cózar A (2018). Characterization of microplastic litter from oceans by an innovative approach based on hyperspectral imaging. Waste Manag.

[40] Shan J, Zhao J, Zhang Y, Liu L, Wu F, Wang X (2019). Simple and rapid detection of microplastics in seawater using hyperspectral imaging technology. Anal Chim Acta.

[41] Hahn A, Gerdts G, Völker C, Niebühr V (2019). Using FTIRS as pre-screening method for detection of microplastic in bulk sediment samples. Sci Total Environ.

[42] Hufnagl B, Steiner D, Renner E, Löder MGJ, Laforsch C, Lohninger H (2019). A methodology for the fast identification and monitoring of microplastics in environmental samples using random decision forest classifiers. Anal Methods.

[43] da Silva VH, Murphy F, Amigo JM, Stedmon C, Strand J (2020). Classification and quantification of microplastics (<100 μm) using a focal plane array–Fourier transform infrared imaging system and machine learning. Anal Chem.

[44] Weisser J, Beer I, Hufnagl B, Hofmann T, Lohninger H, Ivleva NP (2021). From the well to the bottle: identifying sources of microplastics in mineral water. Water.

[45] Renner G, Schmidt TC, Schram J (2017). A new chemometric approach for automatic identification of microplastics from environmental compartments based on FT-IR spectroscopy. Anal Chem.

[46] Renner G, Sauerbier P, Schmidt TC, Schram Jr. Robust automatic identification of microplastics in environmental samples using FTIR microscopy. Anal Chem. 2019;91(15):9656–64. 10.1021/acs.analchem.9b01095. 10.1021/acs.analchem.9b01095 31287674

[47] Primpke S, Wirth M, Lorenz C, Gerdts G (2018). Reference database design for the automated analysis of microplastic samples based on Fourier transform infrared (FTIR) spectroscopy. Anal Bioanal Chem.

[48] Primpke S, Cross RK, Mintenig SM, Simon M, Vianello A, Gerdts G (2020). Toward the systematic identification of microplastics in the environment: evaluation of a new independent software tool (siMPle) for spectroscopic analysis. Appl Spectrosc.

[49] Zhang J, Tian K, Lei C, Min S (2018). Identification and quantification of microplastics in table sea salts using micro-NIR imaging methods. Anal Methods.

[50] Liu F, Olesen KB, Borregaard AR, Vollertsen J (2019). Microplastics in urban and highway stormwater retention ponds. Sci Total Environ.

[51] Kedzierski M, Falcou-Préfol M, Kerros M-E, Henry M, Pedrotti ML, Bruzaud S (2019). A machine learning algorithm for high throughput identification of FTIR spectra: application on microplastics collected in the Mediterranean Sea. Chemosphere.

[52] Brandt J, Bittrich L, Fischer F, Kanaki E, Tagg A, Lenz R (2020). High-throughput analyses of microplastic samples using fourier transform infrared and raman spectrometry. Appl Spectrosc.

[53] Roscher L, Halbach M, Nguyen MT, Hebeler M, Luschtinetz F, Scholz-Böttcher BM, et al. Microplastics in two German wastewater treatment plants: year-long effluent analysis with FTIR and Py-GC/MS. Sci Total Environ. 2022;817:152619. 10.1016/j.scitotenv.2021.152619. 10.1016/j.scitotenv.2021.152619 34968590

[54] Primpke S, Dias P, Gerdts G (2019). Automated identification and quantification of microfibres and microplastics. Anal Methods.

[55] Schymanski D, Oßmann BE, Benismail N, Boukerma K, Dallmann G, von der Esch E (2021). Analysis of microplastics in drinking water and other clean water samples with micro-Raman and micro-infrared spectroscopy: minimum requirements and best practice guidelines. Anal Bioanal Chem.

[56] Frei S, Piehl S, Gilfedder B, Löder M, Krutzke J, Wilhelm L (2019). Occurrence of microplastics in the hyporheic zone of rivers. Sci Rep.

[57] Schrank I, Löder MGJ, Imhof HK, Moses SR, Heß M, Schwaiger J, et al. Riverine microplastic contamination in southwest Germany: a large-scale survey. Front Earth Sci. 2022;10. 10.3389/feart.2022.794250.

[58] Möller JN, Heisel I, Satzger A, Vizsolyi EC, Oster SJ, Agarwal S (2022). Tackling the challenge of extracting microplastics from soils: a protocol to purify soil samples for spectroscopic analysis. Environ Toxicol Chem.

[59] Teichert S, Löder MGJ, Pyko I, Mordek M, Schulbert C, Wisshak M (2021). Microplastic contamination of the drilling bivalve Hiatella arctica in Arctic rhodolith beds. Sci Rep.

[60] Dong M, She Z, Xiong X, Luo Z (2021). Automated analysis of microplastics based on vibrational spectroscopy: are we measuring the same metrics?. Anal Bioanal Chem.

[61] Kirstein IV, Hensel F, Gomiero A, Iordachescu L, Vianello A, Wittgren HB, et al. Drinking plastics? – Quantification and qualification of microplastics in drinking water distribution systems by µFTIR and Py-GCMS. Water Res. 2021;188:116519. 10.1016/j.watres.2020.116519. 10.1016/j.watres.2020.116519 33091805

[62] Rasmussen LA, Iordachescu L, Tumlin S, Vollertsen J. A complete mass balance for plastics in a wastewater treatment plant - macroplastics contributes more than microplastics. Water Res. 2021;201:117307. 10.1016/j.watres.2021.117307. 10.1016/j.watres.2021.117307 34116293

[63] Rist S, Vianello A, Winding MHS, Nielsen TG, Almeda R, Torres RR, et al. Quantification of plankton-sized microplastics in a productive coastal Arctic marine ecosystem. Environ Pollut. 2020;266:115248. 10.1016/j.envpol.2020.115248. 10.1016/j.envpol.2020.115248 32738600

[64] Roscher L, Fehres A, Reisel L, Halbach M, Scholz-Böttcher B, Gerriets M, et al. Microplastic pollution in the Weser estuary and the German North Sea. Environ Pollut. 2021;288:117681. 10.1016/j.envpol.2021.117681. 10.1016/j.envpol.2021.117681 34284208

[65] Mintenig SM, Kooi M, Erich MW, Primpke S, Redondo- Hasselerharm PE, Dekker SC, et al. A systems approach to understand microplastic occurrence and variability in Dutch riverine surface waters. Water Res. 2020;176:115723. 10.1016/j.watres.2020.115723. 10.1016/j.watres.2020.115723 32220661

[66] Primpke S, Fischer M, Lorenz C, Gerdts G, Scholz-Böttcher B (2020). Comparison of pyrolysis gas chromatography/mass spectrometry and hyperspectral FTIR imaging spectroscopy for the analysis of microplastics. Anal Bioanal Chem.

[67] Abel SM, Primpke S, Int-Veen I, Brandt A, Gerdts G. Systematic identification of microplastics in abyssal and hadal sediments of the Kuril Kamchatka trench. Environ Pollut. 2021;269:116095. 10.1016/j.envpol.2020.116095. 10.1016/j.envpol.2020.116095 33257152

[68] Löder M, Imhof H, Ladehoff M, Löschel L, Lorenz C, Mintenig S (2017). Enzymatic Purification of Microplastics in Environmental Samples. Environ Sci Technol.

[69] Lorenz C, Roscher L, Meyer MS, Hildebrandt L, Prume J, Löder MGJ (2019). Spatial distribution of microplastics in sediments and surface waters of the southern North Sea. Environ Pollut.

[70] Mári Á, Bordós G, Gergely S, Büki M, Háhn J, Palotai Z, et al. Validation of microplastic sample preparation method for freshwater samples. Water Res. 2021;202:117409. 10.1016/j.watres.2021.117409. 10.1016/j.watres.2021.117409 34271455

[71] Westad F, Marini F (2015). Validation of chemometric models – a tutorial. Anal Chim Acta.

[72] Demšar J (2006). Statistical comparisons of classifiers over multiple data sets. J Mach Learn Res.

[73] Primpke S, Godejohann M, Gerdts G (2020). Rapid identification and quantification of microplastics in the environment by quantum cascade laser-based hyperspectral infrared chemical imaging. Environ Sci Technol.

[74] Cabernard L, Roscher L, Lorenz C, Gerdts G, Primpke S (2018). Comparison of Raman and Fourier transform infrared spectroscopy for the quantification of microplastics in the aquatic environment. Environ Sci Technol.

[75] Song YK, Hong SH, Eo S, Shim WJ. A comparison of spectroscopic analysis methods for microplastics: manual, semi-automated, and automated Fourier transform infrared and Raman techniques. Mar Pollut Bull. 2021;173:113101. 10.1016/j.marpolbul.2021.113101. 10.1016/j.marpolbul.2021.113101 34743073

[76] Käppler A, Windrich F, Löder MGJ, Malanin M, Fischer D, Labrenz M (2015). Identification of microplastics by FTIR and Raman microscopy: a novel silicon filter substrate opens the important spectral range below 1300 cm(-1) for FTIR transmission measurements. Anal Bioanal Chem.

