# Supplementary material for: Comparison of two rapid automated analysis tools for large FTIR microplastic datasets
Source: Anal Bioanal Chem. 2023 Mar 20;415(15):2975–87. doi: 10.1007/s00216-023-04630-w (PMC10284987; doi:10.1007/s00216-023-04630-w)
Supplement: Supplementary file 1 — Supplementary file1 (PDF 581 KB) [file 216_2023_4630_MOESM1_ESM.pdf]

Supplementary Data for manuscript titled

Comparison of two rapid automated analysis tools for large FTIR microplastic data sets

Authors: Sonya R. Moses[1]*, Lisa Roscher[2]*, Sebastian Primpke[2+], Benedikt Hufnagl[3,4], Martin G. J. Löder[1+], Gunnar Gerdts[2+#], Christian Laforsch[1+#]

*joint first authorship

#joint senior authorship

[+]corresponding authors:
sebastian.primpke@awi.de
martin.loeder@uni-bayreuth.de
gunnar.gerdts@awi.de
christian.laforsch@uni-bayreuth.de

Affiliations

[1] Department of Animal Ecology I and BayCEER, University of Bayreuth, Universitätsstr. 30, 95440 Bayreuth, Germany

[2] Alfred-Wegener-Institute Helmholtz Centre for Polar and Marine Research, Biologische Anstalt Helgoland, Kurpromenade 201, 27498 Helgoland, Germany

[3] Institute of Chemical Technologies and Analytics, Vienna University of Technology, A 1060 Vienna, Austria

[4] Purency GmbH, Walfischgasse 8/34, A 1010 Vienna, Austria

Content:

Figures

Fig. S 1 Randomly selected PP-spectra (n=10) from siMPle analysis of sample B-06.

Fig. S 2 A. Ten randomly selected EVA spectra, extracted from siMPle analysis (sample set A and B) and manually evaluated. B. EVA reference spectra included in siMPle database.

Fig. S 3 Microscopy image of Anodisc filter with sample B-03 (A) and A-08 (B), showing high amounts of material with biogenic appearance.

Tables

Tab. S 1 Background information on subsamples reanalysed in the present study, originating from samples collected in the catchment area of the River Weser and transition to the North Sea in the framework of previous studies (Roscher et al. (2021), Moses et al. (unpublished data)).

Tab. S 2 MP count of polymer types detected in sample set A after analysis with siMPle/MPAPP. The clusters A/PUR/V, CA and EVA were excluded from analysis. For abbreviations refer to Tab. 1 in main manuscript.

Tab. S 3 MP count of polymer types detected in sample set A after analysis with BPF. The clusters A/PUR/V, CA and EVA were excluded from analysis. For abbreviations refer to Tab. 1 in main manuscript.

Tab. S 4 MP count of polymer types detected in sample set B after analysis with siMPle. The clusters A/PUR/V, CA and EVA were excluded from analysis. For abbreviations refer to Tab. 1 in main manuscript.

Tab. S 5 MP count of polymer types detected in sample set B after analysis with BPF. The clusters A/PUR/V, CA and EVA were excluded from analysis. For abbreviations refer to Tab. 1 in main manuscript.

Tab. S 1 Background information including suspended particulate matter (SPM) on subsamples reanalysed in the present study, originating from samples collected in the catchment area of the River Weser (A-01 – A-10) and transition to the North Sea (B-01 – B-10) in the framework of previous studies (Roscher et al. [1], Moses et al. (unpublished data))

Sample ID	Sampling area	North	East	SPM [mg/L]
A-01	Fulda	50°35.91	09°38.34	3.7
A-02	Eder	51°02.98	08°17.05	1.3
A-03	Eder	51°07.52	09°17.70	1.8
A-04	Fulda	51°19.46	09°31.70	6.6
A-05	Werra	51°24.99	09°40.20	5.5
A-06	Weser	51°25.81	09°38.33	6.0
A-07	Große Aue	52°34.64	09°02.29	9.6
A-08	Leine	52°41.01	09°36.12	16.5
A-09	Örtze	52°44.33	10°02.01	11.5
A-10	Wümme	53°04.54	09°12.33	7.6
B-01	Jade Bay	53°29.94	08°14.18	147.6
B-02	Jade Bay	53°27.87	08°14.00	85.6
B-03	Lower Weser	53°21.86	08°30.19	29.7
B-04	Lower Weser	53°25.46	08°29.88	47.1
B-05	Outer Weser	53°32.74	08°33.43	54.0
B-06	Outer Weser	53°35.22	08°31.12	14.3
B-07	Outer Weser	53°37.75	08°28.01	35.7
B-08	Outer Weser	53°39.27	08°24.32	29.9
B-09	North Sea	53°52.78	07°39.42	0.8
B-10	North Sea	53°59.53	07°50.61	12.8

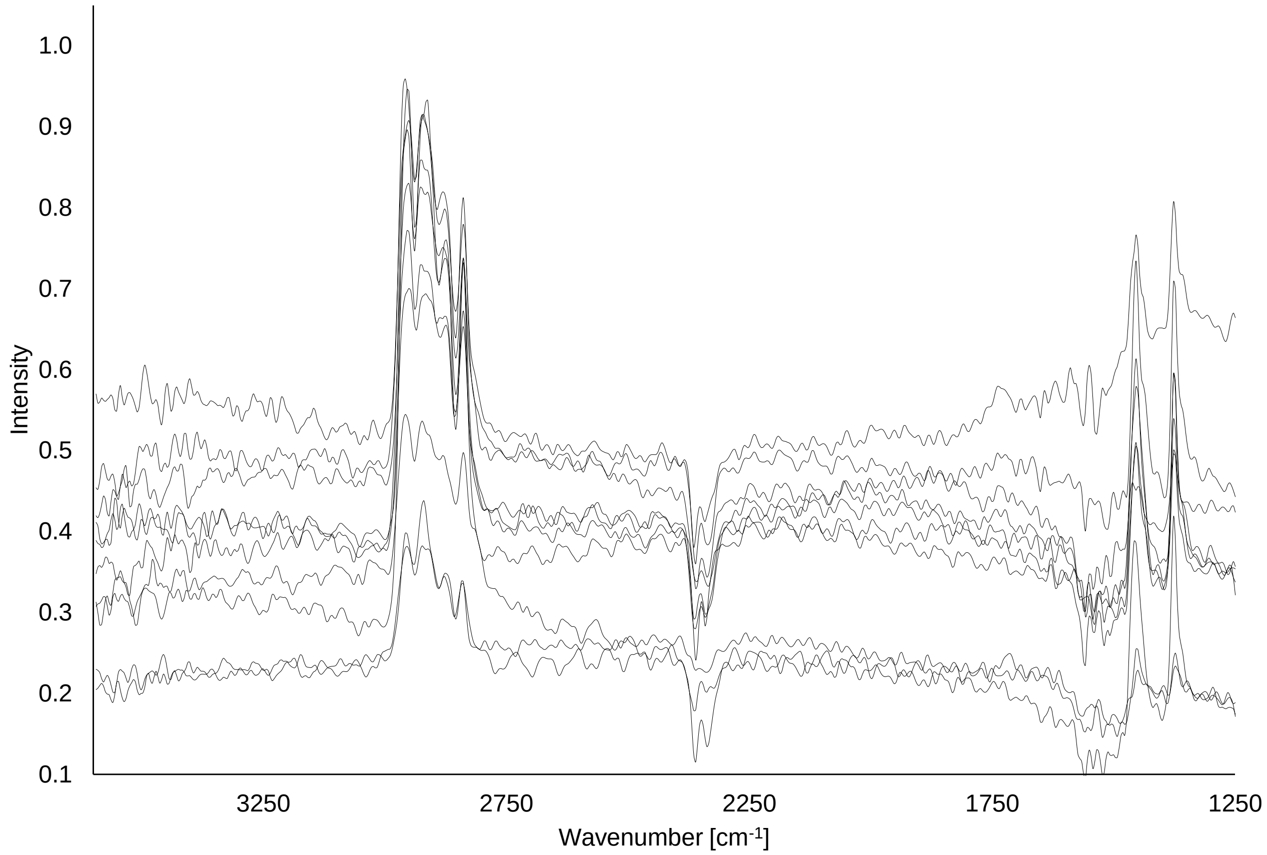

Fig. S 1 Randomly selected PP-spectra (n=10) from siMPle analysis of sample B-06

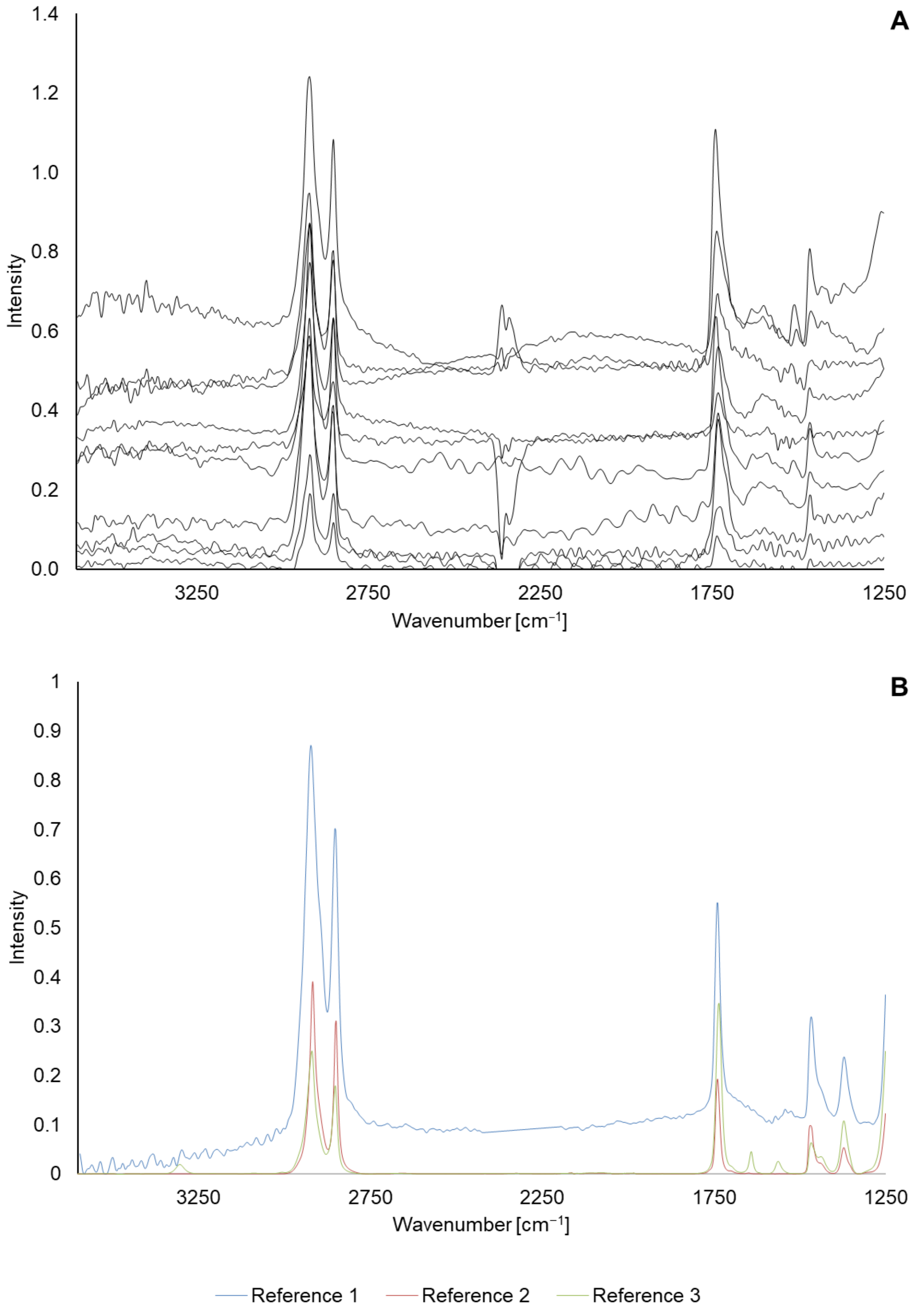

Fig. S 2 A. Ten randomly selected EVA spectra, extracted from siMPle analysis (sample set A and B) and manually evaluated. B. EVA reference spectra included in siMPle database

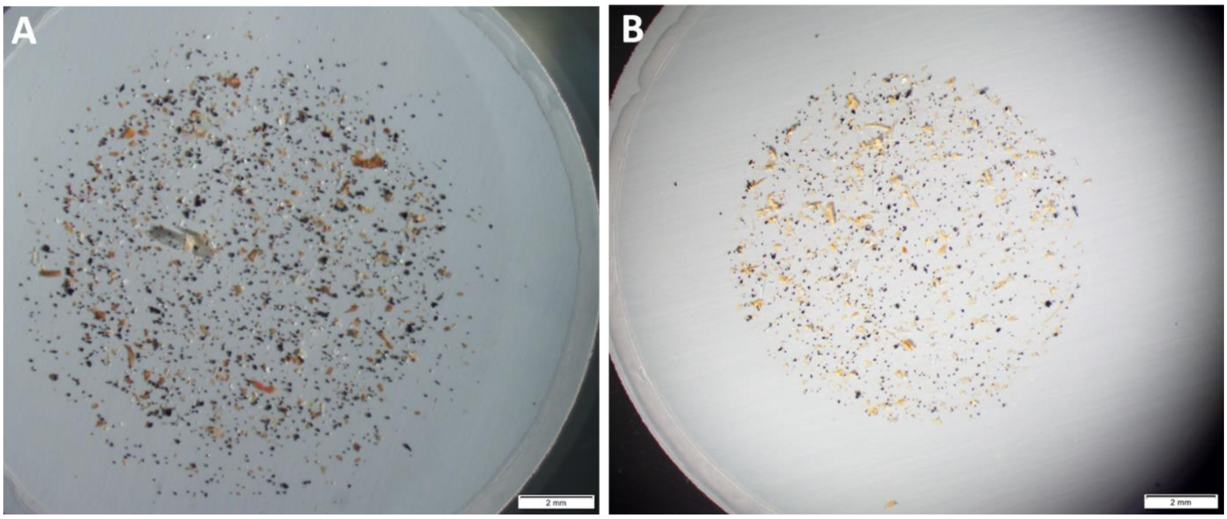

Fig. S 3 Microscopy image of Anodisc filter with sample B-03 (A) and A-08 (B), showing high amounts of material with biogenic appearance

Tab. S 2 MP count of polymer types detected in sample set A after analysis with siMPle/MPAPP. The clusters A/PUR/V, CA and EVA were excluded from analysis. For abbreviations refer to Table 1 in main manuscript

	PE	PP	PS	PC	PA	PVC	CA	PEST	A/PUR/V	PSU	PEEK	EVA	POM	∑	∑ without A/PUR/V	∑ without A/PUR/V, CA and EVA
**A-01**	1	4	0	0	0	0	0	0	91	0	0	4	0	100	9	5
**A-02**	0	1	0	0	0	0	0	1	60	0	0	1	0	63	3	2
**A-03**	0	11	0	0	0	0	0	0	59	0	0	1	0	71	12	11
**A-04**	5	8	1	0	1	0	0	0	85	1	0	2	0	103	18	16
**A-05**	5	9	1	0	2	1	0	4	69	0	0	2	0	93	24	22
**A-06**	6	14	1	0	1	0	0	4	121	0	0	7	0	154	33	26
**A-07**	0	3	0	0	0	0	0	0	6	0	0	0	0	9	3	3
**A-08**	11	76	0	0	4	0	0	5	252	0	0	25	0	373	121	96
**A-09**	0	3	0	0	2	0	0	1	158	0	0	5	0	169	11	6
**A-10**	0	3	0	0	0	0	0	1	185	0	0	8	0	197	12	4
∑	28	132	3	0	10	1	0	16	1086	1	0	55	0	1332	246	191

Tab. S 3 MP count of polymer types detected in sample set A after analysis with BPF. The clusters A/PUR/V, CA and EVA were excluded from analysis. For abbreviations refer to Table 1 in main manuscript

	PE	PP	PS	PC	PA	PVC	CA	PEST	A/PUR/V	PSU	PEEK	EVA	POM	∑	∑ without A/PUR/V	∑ without A/PUR/V, CA and EVA
**A-01**	3	3	0	0	0	0	0	0	3	0	0	0	0	9	6	6
**A-02**	0	2	0	0	0	0	0	0	0	0	0	0	0	2	2	2
**A-03**	0	11	0	0	0	0	0	0	1	0	0	0	0	12	11	11
**A-04**	0	5	1	0	0	0	0	0	0	0	0	0	0	6	6	6
**A-05**	1	10	1	0	0	2	0	0	0	0	0	0	1	15	15	15
**A-06**	2	14	2	0	0	0	0	0	0	0	0	0	0	18	18	18
**A-07**	0	3	0	0	0	1	0	0	0	0	0	0	0	4	4	4
**A-08**	8	47	1	0	3	1	0	9	0	0	0	0	0	69	69	69
**A-09**	0	4	0	0	0	0	0	0	0	0	0	0	0	4	4	4
**A-10**	0	4	0	0	0	1	0	0	0	0	0	0	0	5	5	5
∑	14	103	5	0	3	5	0	9	4	0	0	0	1	144	140	140

Tab. S 4 MP count of polymer types detected in sample set B after analysis with siMPle. The clusters A/PUR/V, CA and EVA were excluded from analysis. For abbreviations refer to Table 1 in main manuscript

	PE	PP	PS	PC	PA	PVC	CA	PEST	A/PUR/V	PSU	PEEK	EVA	POM	∑	∑ without A/PUR/V	∑ without A/PUR/V, CA and EVA
**B-01**	3	2	4	0	1	0	0	2	15	0	0	0	0	27	12	12
**B-02**	1	0	1	0	14	0	0	1	10	0	0	0	0	27	17	17
**B-03**	14	14	3	0	20	0	0	2	393	0	0	39	0	485	92	53
**B-04**	7	21	9	0	2	1	0	1	78	1	0	3	0	123	45	42
**B-05**	3	5	6	1	6	0	0	1	53	6	1	4	0	86	33	29
**B-06**	1	9	2	0	1	0	0	1	13	3	0	1	0	31	18	17
**B-07**	5	12	4	0	0	3	0	0	61	0	0	7	0	92	31	24
**B-08**	1	4	1	0	1	0	0	2	60	0	0	1	0	70	10	9
**B-09**	0	0	0	0	1	1	0	0	7	0	0	0	0	9	2	2
**B-10**	0	0	1	0	1	0	14	1	326	0	0	0	0	343	17	3
∑	35	67	31	1	47	5	14	11	1016	10	1	55	0	**1293**	**277**	**208**

Tab. S 5 MP count of polymer types detected in sample set B after analysis with BPF. The clusters A/PUR/V, CA and EVA were excluded from analysis. For abbreviations refer to Table 1 in main manuscript

	PE	PP	PS	PC	PA	PVC	CA	PEST	A/PUR/V	PSU	PEEK	EVA	POM	∑	∑ without A/PUR/V	∑ without A/PUR/V, CA and EVA
**B-01**	0	0	0	0	0	0	0	0	5	0	0	0	0	5	0	0
**B-02**	0	0	0	0	0	0	0	1	5	0	0	0	0	6	1	1
**B-03**	0	0	0	0	0	0	0	1	42	0	0	0	0	43	1	1
**B-04**	7	16	9	0	0	2	0	2	3	1	0	0	0	40	37	37
**B-05**	1	0	0	0	12	2	0	0	10	1	0	0	0	26	16	16
**B-06**	7	0	0	0	2	0	0	0	2	1	0	0	0	12	10	10
**B-07**	17	0	0	0	0	2	0	0	11	0	0	0	0	30	19	19
**B-08**	0	0	0	0	1	0	0	3	6	0	0	0	0	10	4	4
**B-09**	0	0	0	0	0	0	0	0	9	0	0	0	0	9	0	0
**B-10**	0	0	0	0	0	0	0	0	15	0	0	0	0	15	0	0
∑	32	16	9	0	15	6	0	7	108	3	0	0	0	196	88	88

References supplementary

1. Roscher L, Fehres A, Reisel L, Halbach M, Scholz-Böttcher B, Gerriets M, et al. Microplastic pollution in the Weser estuary and the German North Sea. Environ Pollut. 2021;288:117681. https://doi.org/10.1016/j.envpol.2021.117681
